# Effects of Aloe-Emodin on the Expression of Brain Aquaporins and Secretion of Neurotrophic Factors in a Rat Model of Post-Stroke Depression

**DOI:** 10.3390/ijms24065206

**Published:** 2023-03-08

**Authors:** Yang Liu, Jing Peng, Qinjie Leng, Yang Tian, Xiaoqing Wu, Rui Tan

**Affiliations:** College of Life Science and Engineering, Southwest Jiao tong University, Chengdu 610031, China

**Keywords:** post-stroke depression, aloe-emodin, BDNF, NTF3, aquaporins, GFAP, TRPV4

## Abstract

Post-stroke depression (PSD) is a common complication of stroke that can damage patients’ brains. More and more studies have been conducted on PSD in recent years, but the exact mechanism is still not understood. Currently, animal models provide an alternative approach to better understand the pathophysiology of PSD and may also pave the way for the discovery of new treatments for depression. This study investigated the therapeutic effect and mechanism of aloe-emodin (AE) on PSD rats. Previous studies have shown that AE positively affects PSD in rats by improving depression, increasing their activities and curiosities, enhancing the number of neurons, and ameliorating damage to brain tissue. Meanwhile, AE could up-regulate the expression of brain-derived neurotrophic factor (BDNF) and neurotrophic 3 (NTF3), but it could also down-regulate the expression of aquaporins (AQP3, AQP4, and AQP5), glial fibrillary acidic protein (GFAP), and transient receptor potential vanilloid 4 (TRPV4), which is helpful in maintaining homeostasis and alleviating encephaledema. AE may be a prospective solution in the future for the treatment of PSD patients.

## 1. Introduction

Post-stroke depression (PSD), which is a psychological disorder, usually happens to paralytic patients, which is one of the main reasons why patients [1,2] suffer so heavily from the recovery of motor and cognitive function that they are more likely to face relapse or even death [3]. PSD occurs in approximately 25–30% of ischemic stroke survivors and significantly increases mortality and hypofunction after stroke [4]. However, the available treatments for stroke and PSD are far from satisfactory, and the specific mechanisms of PSD remain unclear. Various theories have been proposed, including psychological stress, neurotransmitter, and inflammatory factor theories [5]. However, these theories mainly focus on the clinical manifestations of patients, with very little basic research on PSD in animal or cellular models and the pathogenesis of PSD [6,7,8,9].

In brain tissue, brain-derived neurotrophic factor (BDNF) and neurotrophic 3 (NTF3) bind with their respective receptors [10], tyrosine kinase receptor B (Trk B) and tyrosine kinase receptor C (Trk C), to activate complex signal transduction pathways, including neurogenesis and synaptic transmission [11,12]. They play an important neuroprotective role in the neurobiological processes involved in mood and anxiety disorders. Numerous studies have shown [13,14,15] that the expression of BDNF and NTF-3 are key targets related to the pathophysiology of depression and anxiety disorders.

Astrocytes are the most abundant cells in the central nervous system (CNS) [16] and play a key role in maintaining water homeostasis; they show the most significant swelling of all cell types during cerebral edema in pathological conditions of the CNS [17,18]. Glial fibrillary acidic protein (GFAP), as the best specific marker protein for astrocyte morphology, exists in most CNS astrocytes [19], which may reflect the reactivity and remodeling of astrocytes [20]. As the most studied astrocyte, AQP4 is crucial to the mediation of water influx during the manifestation of edema as well as the regulation of water efflux during clearance [21]. It is involved in brain edema caused by ischemia and other injuries, as well as in reactive astrocyte migration and glial scarring that occur after traumatic brain injury. AQP1, 3, 4, 5, 8, and 9 have been reported to be expressed to some extent in the brain [22,23], but the functions of AQP3 and 5 remain unclear. The co-expression of aquaporins in astrocytes results in cell swelling and then activates the transient receptor potential vanillin 4 (TRPV4) channels with a quick response to increased cell volume. TRPV4 is an important regulator of cell volume change [24], which can be visually reflected by the changes in the degree of cell swelling. To date, antidepressant treatment does not target PSD yet, and there is not enough knowledge to understand the association between stroke and PSD [25].

Aloe-emodin (AE, 1, 8-dihydroxy-3-hydroxymethyl anthraquinone) is a naturally occurring anthraquinone derived from aloe or rhubarb [26]. It has a variety of pharmacological properties, including anti-cancer, anti-inflammatory, and antioxidant properties, immunosuppression, and neuroprotection [27], and plays an active role in many diseases. A large number of studies [28,29,30] have shown that AE can treat cerebral ischemia and neurodegenerative diseases in anti-inflammatory, anti-apoptotic, and other ways. However, to our knowledge, the pharmacological mechanism of AE in neuroprotection and post-stroke depression treatment has not been investigated. In this study, middle cerebral artery occlusion (MCAO) was combined with chronic unpredictable mild stress (CUMS) to induce a PSD rat model, which was used to investigate the dual therapeutic effects of AE on PSD rats with regard to neuroprotection and reduction in brain swelling, as well as to find the relationship between stroke and PSD in order to understand the mechanism of action of AE against PSD.

## 2. Results

### 2.1. Effect of AE on Depressive-Like Behaviors in Rats

**Figure 1:**
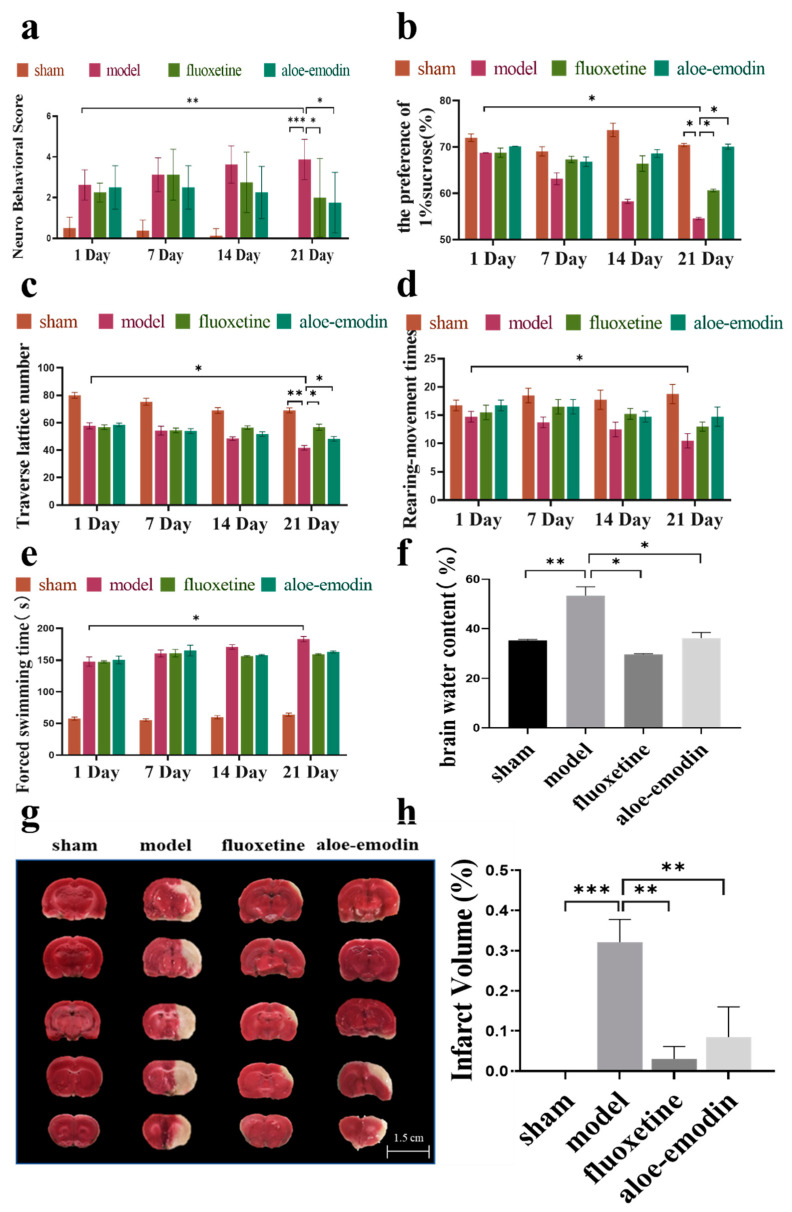
(**a**) Neurofunctional score; (**b**) SPT was used to estimate animal hedonic capacity by measuring sucrose solution intake; (**c**) FST was used to measure animal sensitivity to negative emotions under the threat of drowning; (**d**,**e**) OFT movement reflects reduced animal activity and curiosity; (**f**) brain water content was used to measure the degree of swelling of brain tissue; and (**g**,**h**) TTC staining was used for infarct size detection and quantitative analysis. * *p* < 0.05, ** *p* < 0.01, *** *p* < 0.001.

Neurological function and behavioral scores were used to monitor the depressive status of the rats. Neurofunctional score (NDS) can directly reflect the recovery of neural function in MCAO rats: the lower the score, the better the recovery of a rat. As shown in Figure 1a, with the onset of the depression model, the score of neurological function for the rats in the model group is higher on day 21 compared to day 1 (** *p* < 0.01), while the sham and other administration groups show a downward trend. On day 21 of administration, the neurofunctional scores in the sham, positive-drug, and aloe-emodin groups are significantly lower than those in the model group (* *p* < 0.05), (** *p* < 0.01).

The sucrose preference test (SPT) can assess an animal’s hedonic capacity by measuring the amount of sucrose solution consumed. The more sugar water a rat takes, the better it recovers. As shown in Figure 1b, the rats’ preference for sucrose in the model group decreases on day 21 compared to day 1 (* *p* < 0.05), while the sham and other administration groups show an increasing trend. On day 21 of administration, the rats’ preference for sucrose in the sham group, the positive-drug group, and the aloe-emodin group increases compared to the model group (* *p* < 0.05).

Forced swimming tests (FST) measure the sensitivity of animals to negative emotions under the threat of drowning. The more negative emotions a rat has, the longer it stays still while swimming. As shown in Figure 1c, the time that the rats in the model group keep quiescent while being forced to swim increases on day 21 of administration compared to day 1, while the sham and other administration groups show a downward trend (* *p* < 0.05).

Movement reduction in the open field test (OFT, the number of grids crossed, the times of vertical stands) reflects reduced activity and curiosity, which are common symptoms of depression. As shown in Figure 1d,e, the number of grid crossings and the times of vertical standings of the rats in the model group decrease on day 21 of administration compared to day 1. On day 21 of administration, when compared to the model group, the number of crossing cells in the sham group, the positive-drug group, and the aloe-emodin group increases (* *p* < 0.05), (** *p* < 0.01).

The above results may indicate that AE could achieve the same effect as positive drugs that promote nerve function reparation, improving the activity and curiosity of rats, reducing their sensitivity to negative emotions, and relieving their symptoms of depression after cerebral ischemia.

### 2.2. Cerebral Infarct Size and Cerebral Water Content

Cerebral water content was used to evaluate the severity of brain edema in the rats after having a stroke. As shown in Figure 1f, when compared to the model group, the sham group, the positive-drug group, and the aloe-emodin group show reduced brain water content (** *p* < 0.01).

TTC staining can detect the area of cerebral infarction in rats, as shown in Figure 1g,h. Compared to the model group, the sham group, the positive-drug group, and the aloe-emodin group show a significant reduction in the area of cerebral infarction (** *p* < 0.01).

### 2.3. H&E and Nissl Staining

**Figure 2:**
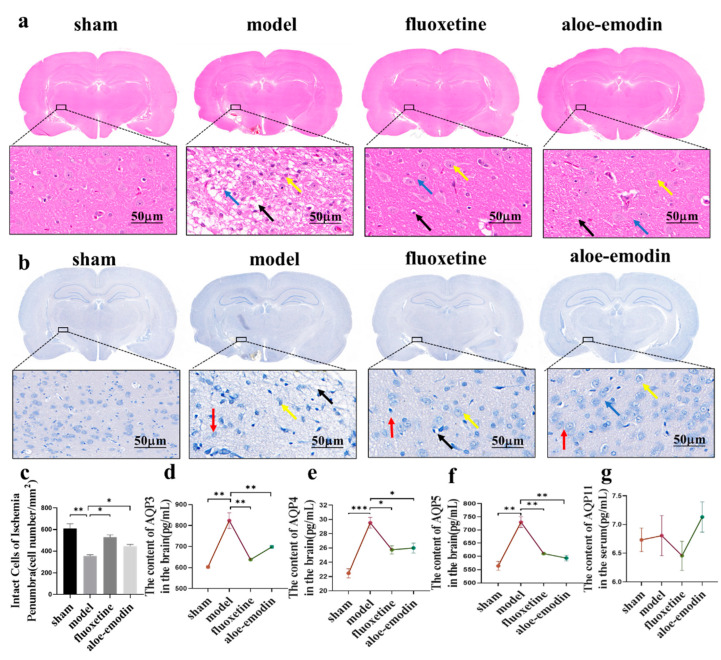
(**a**) Brain tissue slice stained with H&E staining; (**b**) brain tissue slice stained with Nissl staining; (**c**) quantitative analysis of Nissl bodies (n = 3); (**d**) ELISA detection of AQP3 expression in the rats’ brain tissue (n = 3); (**e**) ELISA detection of AQP4 expression in the rats’ brain tissue (n = 3); (**f**) ELISA detection of AQP5 expression in the rats’ brain tissue (n = 3); and (**g**) ELISA detection of AQP3 expression in the rats’ brain tissue (n = 3). * *p* < 0.05, ** *p* < 0.01, and *** *p* < 0.001.

H&E staining was used to observe the structural damage of the ischemic penumbra in the brain tissue after cerebral ischemia. Figure 2a shows that in the sham group, the cells are full and tightly arranged, and the cell structure is intact. In contrast, in the model group, there is an increase in cell gap (black arrow), along with obvious cavities (blue arrow), tissue structure damage, cell swelling and fragmentation (yellow arrow), and unclear structure. Compared to the model group, the aloe-emodin group and the positive-drug group show a clear brain structure. The cell gap is significantly reduced (black arrow), the cavity size is decreased (blue arrow), and the cells are intact (yellow arrow). The recovery is close to that of the sham group. These results indicate that AE has a significant repairing effect on brain damage in PSD rats.

Cerebral ischemia-reperfusion injury can lead to neuronal apoptosis in a pathological environment, thereby exacerbating PSD. Nissl staining can be used to observe the number of Nissl bodies and the number of neurons in the ischemic penumbra. As shown in Figure 2b,c, in the sham group, there is a large number of Nissl bodies that are tightly arranged and have a dark color, while in the model group, the number of Nissl bodies is significantly reduced (red arrow), the contracted edge disappears (black arrow), and obvious cavities and fissures appear (yellow arrow). Compared to the model group, the aloe-emodin group and the positive drug group show an increase in the number of Nissl bodies (red arrow) (** *p* < 0.01) (* *p* < 0.05), intact structure (black arrow), and smaller cell gaps (yellow arrow). Therefore, the neuronal apoptosis in the PSD rats is reduced, and the neuronal function damage is alleviated.

### 2.4. ELISA

ELISA was used to detect the expression of aquaporins (AQP3, AQP4, AQP5, and AQP11) in the PSD rats’ brain tissue. As shown in Figure 2d–g, compared to the model group, the expressions of AQP3, AQP4, and AQP5 in the brain tissue of the rats in the sham group, the positive-drug group, and the aloe-emodin group are all decreased (** *p* < 0.01) (*** *p* < 0.001) (* *p* < 0.05). Moreover, the effect of AE is similar to that of the positive drug. Compared to the model group, although the expression of AQP11 in the brain tissue of the rats in each drug group is slightly decreased, it is not statistically significant. All of these indicate that AE can reduce the expression of aquaporins (AQP3, AQP4, and AQP5) in PSD rats’ brain tissue and alleviate the damage status of depressed rats after cerebral ischemia.

### 2.5. Immunohistochemical

**Figure 3:**
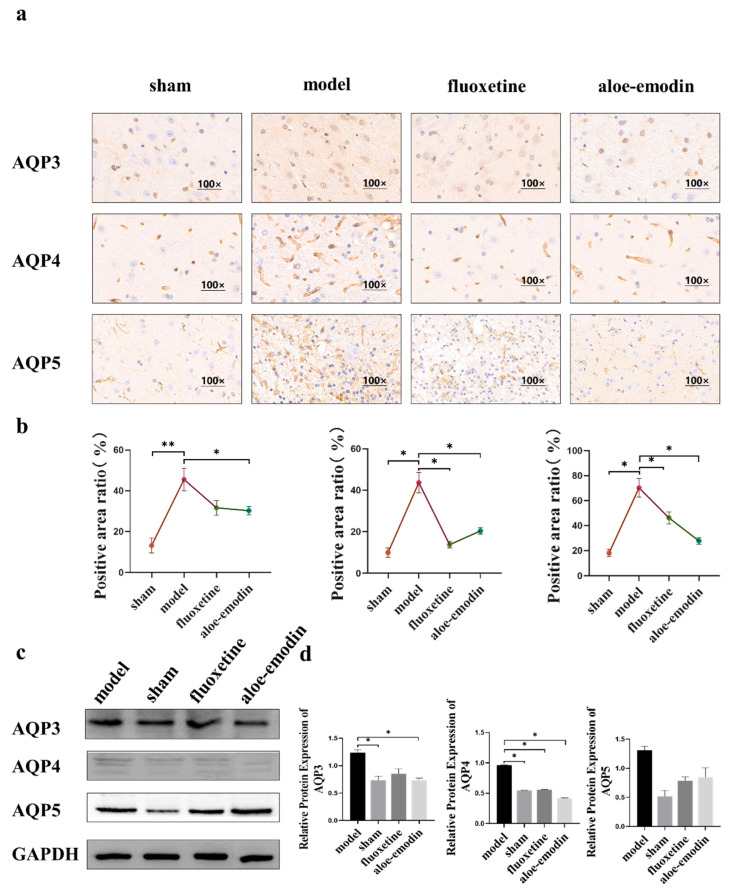
(**a**) Immunohistochemical detection of AQP3, AQP4, and AQP5 expressions in brain tissue; (**b**) quantitative analysis of immunohistochemistry (n = 3); (**c**) WB detection of AQP3, AQP4, and AQP5 protein expressions in brain tissue; (**d**) quantitative analysis of AQP3, AQP4, and AQP5 protein expressions in brain tissue by WB (n = 3). (** *p* < 0.01) (* *p* < 0.05).

For rat brain tissue sections, immunohistochemical staining was performed using AQP3, AQP4, and AQP5 antibodies. By using positive staining grayscale analysis, the area ratio of positive reaction of the brown-stained part in the ischemic penumbra was analyzed. As shown in Figure 3a,b, compared to the model group, the sham group, the positive-drug group, and the aloe-emodin group all show reduced AQP3, AQP4, and AQP5 antibody positive reactions (** *p* < 0.01) (* *p* < 0.05).

**Figure 4:**
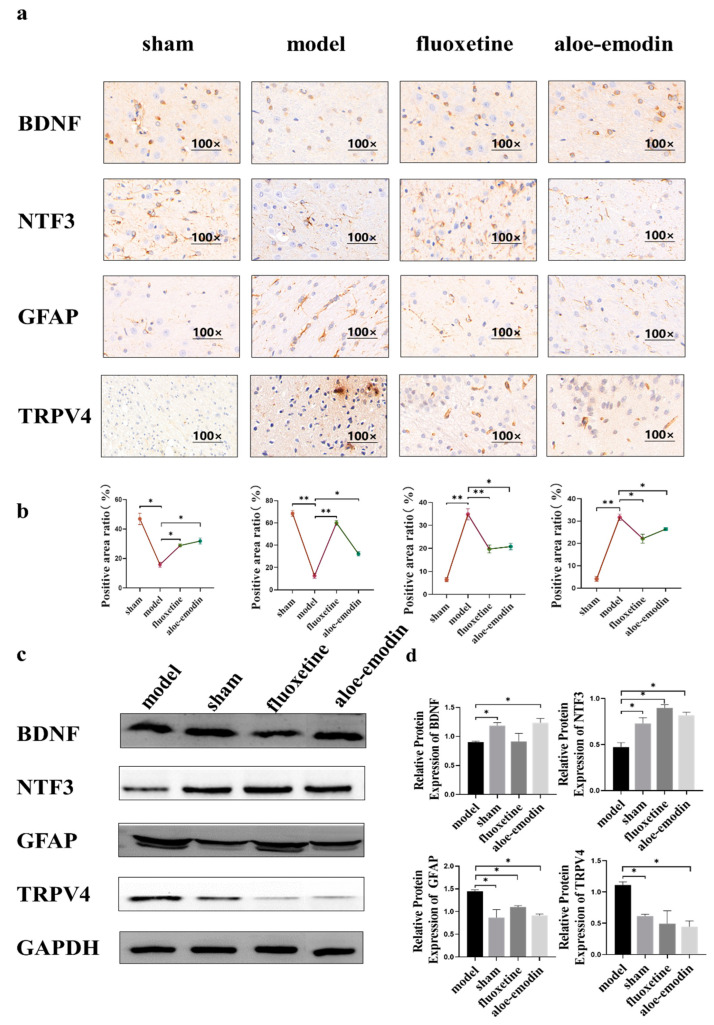
(**a**) Immunohistochemical detection of the expressions of BDNF, NTF3, GFAP, and TRPV4 in brain tissue; (**b**) quantitative analysis of immunohistochemical staining (n = 3); (**c**) Western blot detection of BDNF, NTF3, GFAP, and TRPV4 protein expressions in brain tissue; and (**d**) quantitative analysis of Western blot detection of BDNF, NTF3, GFAP, and TRPV4 protein expressions in brain tissue (n = 3). (** *p* < 0.01) (* *p* < 0.05).

The brain tissue slices of the rats were subjected to immunohistochemical staining using BDNF, NTF3, GFAP, and TRPV4 antibodies. The positive staining grayscale was analyzed to compare the area ratio of positive reaction of the brown-stained part in the ischemic penumbra. As shown in Figure 4a,b, compared to the model group, the positive reactions of BDNF and NTF4 antibodies in the sham group, the positive-drug group, and the aloe-emodin group increase (** *p* < 0.01) (* *p* < 0.05). However, the positive reactions of GFAP and TRPV4 decrease (** *p* < 0.01) (* *p* < 0.05).

### 2.6. Western Blot

Western blot (WB) can be used to detect protein expression in the brain tissue of rats with PSD. As shown in Figure 3c,d, the protein expression of AQP3, AQP4, and AQP5 in the brain tissue of the rats in each treatment group is decreased compared to the model group. Among them, AE can downregulate the protein expression of AQP3 and AQP4 in the rats’ brain tissue (* *p* < 0.05), and the effect is similar to that of the sham group. However, the regulation of AQP5 in each treatment group is not statistically different.

As shown in Figure 4c,d, compared to the model group, the protein expressions of BDNF and NTF3 in the brain tissue of the rats in each treatment group significantly increase (* *p* < 0.05). Meanwhile, the protein expressions of GFAP and TRPV4 in the brain tissue of the rats in each treatment group significantly decrease (* *p* < 0.05).

## 3. Discussion

PSD may have a detrimental effect on the recovery of stroke patients and form a vicious cycle, reducing patients’ cognitive function and autonomy, and even increasing the risk of suicide [31]. Currently, drug therapy is the preferred treatment for PSD. Antidepressants, such as the selective serotonin reuptake inhibitors (SSRIs) fluoxetine and sertraline, are widely used to treat the core symptoms of PSD [32,33]. However, a high proportion of patients do not show a sufficient antidepressant response after taking antidepressants, which may be due to a poor understanding of the underlying neurobiological mechanisms of PSD.

Previous study suggests that AE can protect the brain from damage and exert antioxidant and anti-neuroinflammatory effects by activating the PI3K/AKT/mTOR and NF-κB pathways [34]. However, whether aloe-emodin has neuroprotective effects on PSD rats and improves their neurological function has not been reported yet. A valid animal model of PSD was carefully selected to study the effect and potential mechanism of AE on PSD. Currently, more than 10 animal models of PSD have been generated to advance our understanding of the biology of this disease, of which the combination of CUMS and isolation after ischemia is the most commonly used type of PSD model [35,36]. Pathological reactions, such as neuronal metabolic disorder, relevant inflammatory reaction, neuron loss, and apoptosis, caused by the cerebral infarction are prone to induce a secondary physical injury on patients, thus aggravating the disability and further exacerbating the experience of psychological stress [37]. Therefore, the pathological mechanisms of PSD are complex. The “MCAO + CUMS” model is a typical dual modeling method for cerebral ischemia with superimposed depression, which has a strong face validity and is considered to be a representative PSD model.

Therefore, this study investigated the therapeutic effect of AE on PSD rats by regulating both mood disorders and reducing brain damage. The results showed that AE improved the neurological function and depressive symptoms of PSD rats and produced neuroprotective effects, providing new insights into the relationship between stroke and PSD.

It has been reported that neurotrophic factors are closely related to the condition and prognosis of PSD. BDNF is a type of neurotrophic factor that is involved in the survival and plasticity of neurons. BDNF and NTF3 are widely distributed in the central nervous system and are crucial for the growth and differentiation of the nervous system [2]. BDNF and NTF3 exert their effects on PSD rats by binding to Trk B and Trk C [38]. The main site where nutrients interact with these receptors is the immunoglobulin-like domain of the proximal membrane, which triggers receptor dimerization and tyrosine residue phosphorylation in the cytoplasmic domain [39,40], and activates kinase phosphorylation. Trk receptors act as scaffold molecules and recruit interface proteins, such as protein kinase domain (Src) homologous protein and gelatinous (Shc) adapter proteins. They couple with the receptors to activate downstream signaling pathways, such as extracellular signal-regulated kinase (ERK), phosphatidylinositol 3-kinase (PI3K), mitogen-activated protein kinase (MAPK), and phospholipase C-γ (PLC-γ), thereby modifying gene expression and neural function [41]. BDNF promotes the survival of neurons, increases synaptic plasticity and neurogenesis [42], regulates mood disorders, and alleviates depressive symptoms.

After cerebral ischemia occurs, astrocytes undergo changes, such as cell body enlargement, cell swelling, increased protrusions, and proliferation [43], and the expression of the specific marker GFAP for astrocytes also increases [44]. Under normal physiological conditions, the astrocyte membrane contains various ion channels, AQP4, amino acid transporters, and enzymes to maintain internal homeostasis [45]. When a large amount of ions and neurotransmitters are ingested, the cell’s osmotic pressure increases, causing water to enter the cell and temporarily increase the cell volume, thereby activating the astrocyte volume regulation mechanism [46,47]. The increased cell volume triggers a series of physiological responses, releasing various substances and water to restore normal cell volume and protect neurons [48]. TRPV4 is a non-selective ion channel, mainly functioning as a Ca^2+^ channel, which can act as an osmotic sensor to respond to cell reactions in the brain and regulate changes in osmotic pressure in the brain [49,50]. When cerebral ischemia occurs, TRPV4 channels are activated and remain open, and Ca^2+^ enters cells through these TRPV4 channels, increasing the cell’s osmotic pressure and causing water to enter the cell through the water channel protein, ultimately leading to cell swelling and Ca^2+^ overload [51]. The large influx of extracellular Ca^2+^ triggers the massive release of Glu at the synaptic terminals, and the release rate is much higher than the uptake rate, leading to the accumulation of extracellular Glu [50]. Excessive release of Glu can produce strong cell toxicity, causing a series of toxic reactions and leading to further neurodegenerative diseases.

**Figure 5:**
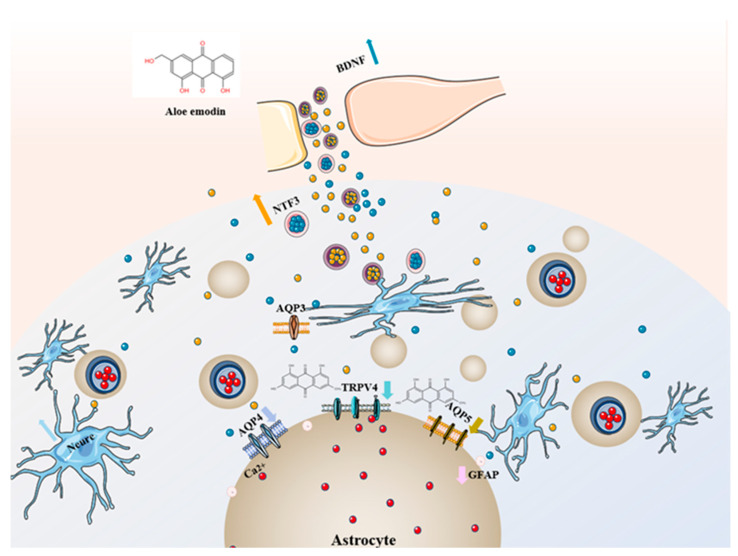
Mechanism of action of aloe emodin.

In summary, As shown in Figure 5, based on the above research findings and combined with the results of the experiment, this study shows that aloe-emodin can promote the secretion of nerve growth factors BDNF and NTF3, providing favorable conditions for the growth and survival of brain neurons and regulating emotional disorders. On the other hand, by inhibiting the expression of TRPV4, it further suppresses Ca^2+^ influx into cells, lowers the Glu level in brain tissue, inhibits the expression of aquaporins, reduces water entry into astrocytes after cerebral ischemia, improves cell swelling, and treats cerebral ischemic edema. We hope that the dual therapeutic effects of AE on PSD can provide a new pharmacological basis for its clinical application in PSD patients.

## 4. Materials and Methods

### 4.1. Animals

Fifty male SD rats of SPF grade were used in the experiment, with a weight of (260 ± 10) g, and the animal license number was SCXK (Chuan) 2020–2030, as provided by Chengdu Dashuo Biotechnology Co., Ltd. (Binzhou, China). The animals were housed at Southwest Jiaotong University at a temperature of 24–26°C, a relative humidity of 40–60%, and a 12 h light/dark cycle, with free access to food and water. The animals were acclimated for one week prior to the experiment. All operations in this experiment complied with the requirements of the Southwest Jiaotong University Experimental Animal Ethics Committee, with approval number SWJTU-2020-0824-05.

### 4.2. Reagents

The reagents used in the experiment were aloe-emodin (BJS9141396, Picasso, Shanghai, China), fluoxetine hydrochloride (J20130010, Patheon, Suzhou, China), SDS-PAGE gel preparation kit (G2003-50T, Servicebio, Suzhou, China), PVDF membrane (IPVH00010, Millipore of USA MA), Allergic ECL Chemiluminescence substrate (BL520B, Biosharp, Anhui, China), Beta Actin-Ab (AF7018, Affinity, Jangsu, China), goat anti-rabbit secondary antibody -HRP (70-GAR0072, Multi sciences, Hangzhou, China), GAPDH antibodies (AF7021, Affinity, Jangsu, China), AQP3 antibodies (AF5222, Affinity, Jangsu, China), AQP4 antibodies (AF5164, Affinity, Jangsu, China), AQP5 antibodies (AF5169, Affinity, Jangsu, China), BDNF antibodies (ab108319, Abcam, Shanghai, China).

### 4.3. Middle Cerebral Artery Occlusion Model

Male SD rats weighing between 280–300 g were selected for the study. The MCAO model was constructed using the suture method [52]. After anesthesia, the rats were fixed on a surgical board. A midline incision was made in the neck, and the subcutaneous tissue was bluntly separated to expose the right common carotid artery, external carotid artery, and internal carotid artery. The proximal end of the right common carotid artery was ligated, and a specially designed suture was inserted through the cut end and slowly pushed in until it encountered resistance (suture depth was 20 ± 2 mm), causing obstruction of the right brain of the rat. The incision was sutured, and penicillin was injected into the abdomen to prevent infection. The suture was removed after 2 h of ischemia to allow reperfusion. The Zea–Longa [53] scoring system was used to evaluate the degree of neurological functional deficits in the rats. As shown in Table 1, the rats with Zea–Longa scores ≥1 were considered successfully modeled and could be included in subsequent experiments. The rats with scores of 4 or 5 were not included in the experiment due to the severity of their brain infarction.

### 4.4. Post-stroke Depression Model

At 7 days after the MCAO modeling, CUMS was administered for 21 days [54]: the rat cages were tilted at a 45-degree angle, the rats’ behavior was restricted for 2 h, water and food were withheld for 24 h, moist bedding was provided for 24 h, the tails were clipped for 1 min, and the day–night cycle was reversed. One type of stimulus was selected each day, and different stimuli were used on consecutive days. The sham surgery group received no treatment.

### 4.5. Groups and Drug Administration

**Figure 6:**
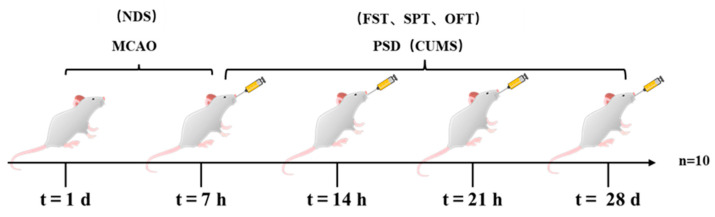
Drug administration in rats.

As shown in Figure 6, thirty male SD rats with successful MCAO modeling were randomly divided into four groups: model group, positive-drug (fluoxetine) group (10 mg/kg) [55], aloe-emodin group (100 mg/kg) [56], and sham group. The sham group underwent the same surgical procedures as the other groups, but without MCAO or PSD modeling. The model group and the sham group received daily oral gavage of saline solution, while the positive-drug group and the aloe-emodin group received daily oral gavage of fluoxetine and aloe-emodin, respectively.

### 4.6. Behavioral Tests

#### 4.6.1. Neurological Function Test

Neurological function damage was evaluated using the Zea-Longa 5-point scale [57]. Neurobehavioral assessments were performed and recorded on days 1, 7, 14, and 21 after the PSD modeling for each group of rats.

#### 4.6.2. Sucrose Preference Test

On the first day, two bottles of 1% sucrose solution were given to each cage of rats. On the second day, food and water were withheld. On the third day, one bottle of regular water and one bottle of 1% sucrose solution were given to each cage of rats. The positions of the two water bottles were exchanged every hour, and the consumption of each bottle was recorded after 3 h. The experimental data were collected on the first, seventh, fourteenth, and twenty-first day after the PSD modeling [58].

Sucrose consumption rate = Sucrose consumption/(Sucrose consumption + Water consumption) × 100%

#### 4.6.3. Open Field Test

The rats were placed in a 70 cm × 70 cm cardboard box in a dark environment. The box was divided into 25 areas using a marker pen, and the number of areas crossed and the number of times the rats stood up were recorded for 1 h. The experimental data were collected on the first, seventh, fourteenth, and twenty-first day after the PSD modeling [59].

#### 4.6.4. Forced Swimming Test (FST)

The rats were placed in a bucket with a diameter of 50 cm and a water depth of 25 cm. After a 1 min adaptation period in the water, the immobility time of the rats was recorded for the following 6 min. The experimental data were collected on the twenty-first day after the PSD modeling [60].

### 4.7. Histopathological Examination

#### 4.7.1. Cerebral Infarct Volume

The brain tissue was collected and frozen at −20 ℃, and then cut into 5 slices. After staining with a TTC solution in the dark, the tissue was fixed with 4% paraformaldehyde. The red area represents the normal brain tissue, and the white area represents the area of cerebral infarction.

Cerebral infarction volume (%) = (M white area/M whole brain) × 100%.

#### 4.7.2. Cerebral Water Content

The fresh brain tissue of the rats was washed, wiped clean, and weighed (wet weight), and then the brain tissue was dried in an oven at 37 ℃ to a constant weight and weighed again (dry weight).

Brain water content (%) = (M wet weight−M dry weight)/M wet weight × 100%

#### 4.7.3. Hematoxylin and Eosin Staining

The rat brain was fixed, dehydrated, cleaned with xylene, embedded in paraffin, and sliced. Hematoxylin and eosin (H&E) staining was performed, followed by 1% hydrochloric acid differentiation, ammonia water bluing, eosin counterstaining, water washing, gradient dehydration, drying, and slide sealing. The samples were then observed under a microscope.

#### 4.7.4. Nissl Staining for Neuropathological Evaluation

Similar to Section 4.7.3., the rats’ brain tissue was fixed, embedded, sliced, and dewaxed. The samples were then stained with 1% toluidine blue, differentiated with 95% alcohol, gradually dehydrated, and observed under a microscope after slide sealing.

#### 4.7.5. Immunohistochemical

The rats’ brain tissue was paraffin-sectioned, dewaxed, antigen-retrieved, and incubated at room temperature. The tissue sections were then incubated overnight with antibodies against AQP3, AQP4, AQP5, BDNF, GFAP, TRPV4, and NTF3. After incubation with secondary antibodies, DAB staining was performed, followed by counterstaining with hematoxylin, dehydration, transparency, and slide sealing.

### 4.8. ELISA

The brain tissue was taken and homogenized. The expression levels of AQP3, AQP4, AQP5, and AQP11 were determined by following the instructions of an ELISA kit.

### 4.9. Western Blotting

The brain tissue was homogenized in a protein lysis buffer and centrifuged; the protein was then extracted and its concentration was determined. Electrophoresis was performed by transferring the samples to a membrane, blocking with skim milk for 1 h, then adding antibodies against AQP3, AQP4, AQP5, BDNF, GFAP, TRPV4, and NTF3, and finally incubating overnight at 4 ℃. After incubation with the corresponding antibodies at room temperature, the signal intensity of the bands was measured using chemiluminescence, and the grayscale values were analyzed using ImageJ software.

### 4.10. Statistical Analysis

The GraphPad Prism 8.0.1 software was used for statistical analysis. The data were expressed as $\bar{x}$ ± s. Comparisons between multiple groups of data were analyzed using one-way ANOVA. *p* < 0.05, *p* < 0.01, and *p* < 0.001 were considered statistically significant. 

## 5. Conclusions

AE can significantly improve behavioral function, reduce depression levels, improve brain tissue damage and lesions, increase the number of brain tissue neurons, decrease the contents of AQP3, AQP4, AQP5, GFAP, and TRPV4 in the brain and brain tissue, and increase the protein expressions of BDNF and NTF3 in PSD rats. AE may have a significant therapeutic effect on PSD rats.

## Figures and Tables

**Table 1:** Neurological score of rats.

Score	Behavioral State
0	Normal
1	Cerebral ischemia prevents contralateral forelimb extension
2	Failure to extend the contralateral forelimb in cerebral ischemia
3	Pour to the opposite side of the ischemic brain
4	Loss of consciousness, coma
5	Death

## Data Availability

Not applicable.
